# Disordered eating and emotion dysregulation among adolescents and their parents

**DOI:** 10.1186/s40359-017-0180-5

**Published:** 2017-04-04

**Authors:** Erika Hansson, Daiva Daukantaité, Per Johnsson

**Affiliations:** 1 Kristianstad University, Kristianstad, Sweden; 2 Lund University, Lund, Sweden

**Keywords:** Disordered eating, Emotion dysregulation, Adolescents, Parents, Shared meals

## Abstract

**Background:**

Research on the relationships between adolescent and parental disordered eating (DE) and emotion dysregulation is scarce. Thus, the aim of this study was to explore whether mothers’ and fathers’ own DE, as measured by SCOFF questionnaire, and emotion dysregulation, as measured by the difficulties in emotion regulation scale (DERS), were associated with their daughters’ or sons’ DE and emotion dysregulation. Furthermore, the importance of shared family meals and possible parent-related predictors of adolescent DE were explored.

**Method:**

The total sample comprised 1,265 adolescents (*M*_*age*_ = 16.19, *SD* = 1.21; age range 13.5–19 years, 54.5% female) whose parents had received a self-report questionnaire via mail. Of these, 235 adolescents (18.6% of the total sample) whose parents completed the questionnaire were used in the analyses. Parents’ responses were matched and compared with those of their child.

**Results:**

Adolescent girls showed greater levels of DE overall than did their parents. Furthermore, DE was associated with emotion dysregulation among both adolescents and parents. Adolescent and parental emotion dysregulation was associated, although there were gender differences in the specifics of this relationship. The frequency of shared dinner meals was the only variable that was associated to DE and emotion dysregulation among adolescents, while parental eating disorder was the only variable that enhanced the probability of adolescent DE.

**Conclusion:**

The present study contributes to the literature by demonstrating that there are significant associations between parents and their adolescent children in terms of DE, emotion dysregulation, and shared family meals. Future studies should break down these relationships among mothers, fathers, girls, and boys to further clarify the specific associational, and possibly predictive, directions.

## Background

Evidence for the parental influence on adolescents’ eating disorders and disordered eating (DE)-namely, the behaviors and attitudes toward body perception, eating habits, weight regulation, and self-evaluation [46]-is inconclusive at present. Research exploring the specific pathways through which parental behaviors operate in this capacity is needed to clarify why some adolescents develop DE while others do not [22, 47]. Traditionally, responsibility has been attributed to mothers for the occurrence of eating disorders in children (primarily daughters) [11], which has led most studies to explore different aspects of DE among mothers and daughters, such as mothers’ talk about their own and their daughters’ weight with their daughters [4], or the similarity of coping styles between mothers and daughters with eating disorders [25]. Despite this focus on mother-daughter relations, associations between parenting practices and eating behaviors have also been found among adolescent boys [28], making it clear that further efforts to understand the parental influences on boys’ and girls’ DE are needed. This is especially important since the prevalence of DE, which may lead to a clinical eating disorder [35] that renders high morbidity [19, 42] is rather high, varying from 15 to 24% in boys and to 25 to 52% in girls [16–18]. According to Wertheim [47], mothers’ and fathers’ food abstaining behaviors have predicted the food abstaining behaviors of their adolescent daughters but there is insufficient research on adolescents of both genders [28], and thus, the present study sought to fill this gap by exploring in what ways mothers’ and fathers’ DE is associated with that of their adolescent children.

Parental comments on a child’s weight do appear to be the most consistent correlate of child weight and shape concerns and behaviors; however, one study found that children’s modeling of parental weight concerns through observation of parents’ own dieting behavior, weight-related concerns, and weight loss attempts gave rise to DE among children in fourth and fifth grade [43]. Such indirect parental influences suggests that children are influenced by their parents through modelling certain behaviors and the receiving of covert messages that communicate parental beliefs and expectations, as explained by social learning theory [2, 48]. Furthermore, such indirect parental influences, might have a stronger effect in adolescence due to their unobtrusive nature, which is especially relevant when considering adolescents’ increased independence from parental control with age [37, 49].

DE has also been found to relate to emotion dysregulation among adults [50] and adolescents of both genders [30]. Children of both genders have been shown to express their emotions in different ways-girls are more likely to exhibit more positive and internalizing emotions, whereas boys are more likely to exhibit externalizing emotions [8]. However, as with DE, most research on emotion dysregulation has focused on mothers and daughters, finding evidence that children of depressed mothers are more likely to engage in maladaptive emotion regulation strategies than are children of non-depressed mothers [14, 40]. Furthermore, emotion regulation during middle childhood and adolescence appears to be more closely related to the emotion regulation ability of the mother than of the father [3]. There is also evidence that mothers and fathers give unique contributions to their child’s development of emotion regulatory skills by responding differently to their children’s sadness behavior [7].

Overall, research on the relationships between parent and adolescent emotion regulation and DE is scarce [3, 21, 32]. As such, the central question examined in this paper was whether parents’ own DE and emotion dysregulation are implicitly associated with their adolescents’. One opportunity for children to observe and model healthy eating behaviors (provided that the eating behaviors of the parents are indeed healthy) is during shared family meals. The number of shared meals has been found to be inversely associated with DE among adolescents [26, 36]; notably, this relationship is most commonly noted among females [41]. It is debated what specific problematic behaviors are reduced by regular shared meals, as some studies have suggested that shared meals serve a protective function against bulimia or extreme weight control behaviors [41]. Evidence does suggest that shared meals serve a protective function for both girls and boys, although in different ways. For example, it appears as if shared meals are associated with a decreased risk of engaging in risk behaviours among girls but not among boys [41].

In summary, to extend the knowledge of factors associated with adolescent DE, we explored the relations between parental and adolescent DE, emotion dysregulation, and shared family meals. We hypothesized that there would be associations as well as differences between parents and adolescents in regards to DE and emotion dysregulation, and that high levels of emotion dysregulation among parents would be associated with high levels of DE among the adolescents. Furthermore, we hypothesized that associations would be found between shared meals, emotion dysregulation, and DE among both adolescents and their parents, and that parental DE, parental eating disorders, parental emotion dysregulation, and shared family meals would affect the probability of DE among the adolescents. The analyses were stratified by gender in order to examine possible gender differences in these relationships.

## Method

### Participants

#### Adolescents

The study was conducted in a municipality in southern Sweden between January and March 2014. The initial sample comprised 1,265 adolescents (*M*_age_ = 16.19, *SD* = 1.21; age range 13.5 – 19 years, 54.5% female); in this study, however, we only analyzed a subsample of 235 adolescents (18.6% of the total sample) whose parents had completed the necessary questionnaires.

### Parents

The parents of the students who attended the participating schools received a letter with a questionnaire that they were asked to complete and return in a pre-paid envelope. Two hundred ninety mainly Swedish (95.1%) parents (83.8% mothers) responded. Of those, 235 could be matched to their children using the personal identity number of the child. The remaining 55 responses could not be matched because of various reasons. An analysis of differences between children whose parents participated and those whose parents did not is presented in the Result section.

### Measures

#### Disordered eating

##### SCOFF

The SCOFF questionnaire [31] comprises five questions concerning eating habits and attitudes toward weight and body shape, as follows (with the SOCFF acronym letters printed in bold): “Do you make yourself sick (vomit) because you feel uncomfortably full?”; “Do you worry that you have lost control over how much you eat?”; “Have you recently lost more than one stone (15 pounds or about 6.8 kg) in a 3-month period?”; “Do you believe yourself to be fat when others say you are thin?”; and “Would you say that food dominates your life?” A threshold of two positive answers was proposed to indicate the probability of an existing eating disorder [29, 31], and this threshold was used in the present study, although it has been challenged with the idea that only one positive answer would be necessary to raise suspicion of DE, at least among adolescents [16]. The SCOFF was translated into Swedish [16] by one of the authors by using the back translation procedure with the help of another Swede competent in English. The reliability of the SCOFF-scale, assessed with Kuder and Richardson’s formula 20 (KR20), was .42 for the adolescents, and .29 for the parents.

##### Eating disorder

Parents were asked to answer the following question: “Do you suffer from, or have you earlier in life suffered from, a clinical eating disorder?” The response options were “yes,” “no,” or “I don’t want to answer.”

#### Emotion regulatory behaviors

##### Emotion regulation

The Difficulties in Emotion Regulation Scale (DERS) [15] is a 36-item self-report questionnaire that assesses the six dimensions of emotion regulation: (a) lack of emotional *awareness* (e.g., “I am attentive to my feelings” (reversed)); (b) lack of emotional *clarity* (e.g., “I have no idea how I am feeling”); (c) impulse *control* difficulties (e.g., “When I’m upset I feel out of control”); (d) difficulties in engaging in *goal* directed behaviors (e.g., “When I’m upset I have difficulties getting work done”); (e) *non-acceptance* of emotional responses (e.g., “When I’m upset, I feel guilty for feeling that way”); and (f) limited access to emotion regulation *strategies* (e.g., “When I am upset, my emotions feel overwhelming”). The items are rated in terms of how frequently each statement applies to them on a five-point Likert scale ranging from 1 (“almost never”) to 5 (“almost always”). The total score for the DERS is calculated by adding all answers and the subscale scores are also added to a total number. The DERS has demonstrated high internal consistency [15], with a Cronbach’s α of .93. In the current study, the Cronbach’s alpha was .94 for the total scale for adolescents (.77 for the parents), .76 (.34) for awareness, .81(.64) for clarity, .85 (.58) for impulsivity, .86 (.60) for goals, .87 (.51) for non-acceptance, and .86 (.65) for strategies.

#### Shared Meals

The adolescents answered three questions on the frequency of shared meals: “How often do the majority of your family members share breakfast/lunch/dinner with you”? Each question was rated on a 7-point Likert-type scale ranging from 1 (“1 day per week”) to 7 (“7 days per week”). The answers were added into a total score of a maximum of 21 meals per week.

### Procedure

#### Adolescents

Both the legal guardians of the students below age 15 and the adolescents themselves, irrespective of age, received written information on the study aims, procedures, and the right to decline participation, and the same information was given orally to the adolescents on the day of data collection as well. The adolescents were also assured of confidentiality. Parents provided their passive consent-that is, they had to sign and return a form if they refused to allow their child to participate in the study. The adolescents consented to participation by completing the questionnaire, which took approximately one hour.

#### Parents

A questionnaire, together with a letter explaining the survey and the right to decline participation, was sent out to all parents. By filling in the questionnaire and sending it back, the respondents gave their informed consent to participate. The parent questionnaire took approximately 20 min  to complete.

### Statistical analysis

All analyses were calculated by use of the IBM SPSS Statistics program, and Pearson’s (*r*), as well as Point-Biserial (*r*_*pb*_) correlation coefficients were used for all correlations. The analyses involving independent group comparisons were calculated with independent t-tests and the Mann-Whitney U-test, while paired-samples t-tests, as well as the McNemar’s test were used to explore the differences between adolescents and their parents. A logistic regression analysis was used to explore which variables among the parents possibly affect adolescent DE. All *p*-values were two-tailed.

## Results

### Preliminary analysis

Overall, 235 parents (86.4% mothers) with matched adolescents (*M*_*age*_ = 16.18, *SD* = 1.20; 56.6% female) participated in this study. Preliminary analyses were conducted to examine whether there were any significant differences in the study variables between the 235 adolescents whose parents had completed the questionnaire and those whose parents had not, and as can be seen in Table 1, no significant differences were found among the two samples.

**Table 1:** The children whose parents participated and children whose parents did not were compared to determine structural differences among the groups

	Parent participated	Parent did not participate	
	*M (SD)* (*Mdn* for SCOFF)	*M (SD)* (*Mdn* for SCOFF*)*	t (u for SCOFF)
SCOFF	0.00	0.00	u = 99716.5, *p =* .07
DERS	73.11 (21.42)	75.19 (23.70)	t (295.59) = 1.11, *p =* .30
*M*_*age*_	16.18 (1.20)	16.19 (1.22)	t (1262) = .084, *p* = .93

*Note*: For each analysis, we excluded the data of adolescents with missing values in a pairwise fashion. Samples whose parents did not participate and whose parents did participate ranged from 642 to 1029 adolescents and 173 to 235 adolescents, respectively

As can be seen from the *p*-values no such significant differences were found

No significant differences were found between mothers and fathers regarding DE, as measured by the SCOFF, or in emotion dysregulation, as measured by the DERS. Therefore, mothers and fathers were not separated in the following analyses.

### Parental and adolescent DE

Parental and adolescent DE was associated among the girls (*r*_*pb*_ = .19, *p ≤ .05*), but not among the boys. As Table 2 shows, about 90% of adolescents and 95% of parents reported fewer than two “yes” answers on the SCOFF and none of the participants answered “yes” to more than three SCOFF questions in total.

**Table 2:** Number of “yes” answers on the SCOFF among adolescents and parents

“Yes” answers	Adolescents (*n*)	Adolescent Girls (*n*)	Adolescent Boys (*n*)	Parents (*n*)	Mothers (*n*)	Fathers (*n*)
0	(164) 73.9%	(78) 63.4%	(86) 86.9%	(190) 81.9%	(163) 81.5%	(27) 84.5%
1	(35) 15.8%	(23) 18.7%	(12) 12.1%	(32) 13.8%	(29) 14.5%	(3) 9.5%
2	(19) 8.6%	(18) 14.7%	(1) 1.0%	(9) 3.9%	(8) 4%	(1) 3%
3	(4) 1.7%	(4) 3.2%	(0)	(1) 0.4%	(0) 0%	(1) 3%

More than two “yes” answers are indicative of an eating disorder

To further illustrate the presence of DE among adolescents and their parents, a McNemar’s chi-square test (Table 3) was conducted, and the results indicated significantly less DE among the parents than among the adolescents (*p* ≤ .05).

**Table 3:** Observed frequencies of DE among adolescents and parents (based on two “yes” answers on SCOFF)

	Adolescents				
	Girls		Boys		
	DE	No DE	DE	No DE	Total
Parents with DE	4 (18%)	5 (5%)	0 (0%)	1 (1%)	10 (4.5%)
Parents without DE	18 (82%)	93 (95%)	1 (100%)	97 (99%)	209 (95.5%)
Total	22 (100%)	98 (100%)	1 (100%)	98 (100%)	219 (100%)

*Note*: The boys’ association was not significant according to McNemar’s test

Five mothers and one father answered “yes” to the question of whether they suffered from, or had earlier in life suffered from, a clinical eating disorder.

### Parental and adolescent emotion dysregulation

An independent *t*-test showed that girls (*M* = 79.49, *SD* = 20.76) tended to have higher total scores on the DERS than did boys (*M* = 65.16, *SD* = 19.60, t (171) = 4.63 *p ≤* .001, Cohen’s *d* = 0.72), which indicates that boys tend to have less emotion dysregulatory problems than girls. Furthermore, there was a significant positive correlation between adolescents’ total DERS scores and their parents’ (*r* = .18, *p ≤* .01). Table 4 shows the means and standard deviations of the girls, boys, and parents for the total and subscale scores of the DERS.

**Table 4:** Girls’, boys’ and parents’ means and standard deviations on the total and subscale scores of the DERS

	Girls		Boys		Parents	
DERS	*M*	*SD*	*M*	*SD*	*M*	*SD*
Total	79.49	20.76	65.16	19.60	58.53	13.52
Awareness	16.27	5.05	15.68	5.37	12.40	4.11
Clarity	11.82	3.98	8.68	3.62	6.86	2.06
Impulse control	11.23	5.25	9.17	4.28	8.24	2.48
Goals	13.13	4.95	10.91	4.51	9.63	3.42
Non-acceptance	12.72	5.40	9.22	3.50	10.04	3.98
Strategies	16.39	6.76	12.72	5.10	11.32	3.28

#### Girls

Paired samples t-tests showed that girls (*M* = 79.49, *SD* = 20.76) significantly differed in DERS total score from their parents (*M* = 58.53, *SD* = 13.52, t (89) = 7.19, p ≤ .001, Cohen’s d = 0.99), indicating that girls experienced greater emotion dysregulation problems than did parents. Parents’ *strategies* subscale score correlated with girls’ scores on the *strategies* (*r* = .25, *p ≤* .01), *impulse-control* (*r* = .19, *p* ≤ .05), and *emotional goals* (*r* = .20, *p* ≤ .05) subscales of the DERS. The parents’ *impulse control* subscale score also correlated with that of girls (*r* = .22, *p* ≤ .05).

#### Boys

Boys (*M* = 65.16, *SD* = 19.60) also showed significantly higher DERS total scores than did their parents (*M* = 58.53, *SD* = 13.52, t (67) = 3.15 *p ≤* .002, Cohen’s *d* = 0.50). Regarding the correlations, parents’ scores on the *clarity* subscale correlated with boys’ scores on the *clarity* (*r* = .27, *p* ≤ .01), *strategies* (*r* = .22 *p* ≤ .05), and *impulse control* (*r* = .30, *p* ≤ .01) subscales. Furthermore, vparents’ scores on the *strategies* subscale significantly correlated with boys’ scores on the *strategies* (*r* = .35, *p* ≤ .01) subscale, while parental *impulse control* scores correlated with boys’ *non-acceptance* (*r* = .24, *p* ≤ .05) and *strategies* (*r* = .27, *p* ≤ .05) scores. Finally, parents’ *non-acceptance* scores correlated with boys’ *non-acceptance* (*r* = .32, *p* ≤ .01) and *strategies* (*r* = .28, *p* ≤ .01) scores.

### The relation between DE and emotion dysregulation among adolescents and parents

DE and emotion dysregulation were significantly correlated in adolescent girls (*r*_*pb*_ = .30, *p ≤ .*01) and in parents (*r*_*pb*_ = .27, *p* ≤ .01), but not in adolescent boys. No significant correlation was found between adolescent DE and parental emotion regulation.

### The relation between family meals, DE, and emotion dysregulation

In regards to shared family meals, no significant correlation was found regarding the total number of meals shared and DE among the adolescents, and neither between the total number of meals shared and emotion dysregulation. However, when only family *dinner* meals were included in the analysis, and the breakfast and lunch meals omitted, weak but significant negative correlations with DE (*r*_*pb*_ = − .16, *p ≤* .01) as well as with emotion dysregulation (*r* = − .16, *p ≤* .05) were found. When exploring girls and boys separately, no significant correlations were observed.

### The effect of parental eating disorders

A logistic regression analysis was performed to assess the impact of a number of factors, including parents’ DE and earlier diagnosed eating disorder, emotion dysregulation, and frequency of shared meals, on the odds that adolescents would report having DE. However, only the results of the girls are presented, as only one boy gave two “yes” answers on SCOFF. The model contained 12 independent variables: eating breakfast, lunch, or dinner together with parent; parental awareness; clarity; impulse control; goals; non-acceptance of emotions; parental emotional strategies; parental SCOFF score; and parental self-reported eating disorder. The full model containing all predictors was significant (for girls) $\chi$ ^*2*^ (1, *N* = 133) = 6.31, *p = .012*, indicating that the model could distinguish the girls who reported DE and girls who did not. The model as a whole explained between 5.7% (Cox and Snell R-square) and 9.3% (Nagelkerke’s R-squared) of the variance in DE and correctly classified 83.2% of the cases. Surprisingly, only one of the independent variables made a significant contribution to the model: whether the parent reported suffering from an eating disorder. The odds ratio was .07, indicating that there is slightly higher odds that girls of parents suffering, or having suffered from an eating disorder would report DE.

## Discussion

In this study the relationships between DE, emotion dysregulation, frequency of shared family meals among adolescents and their parents, as well as possible parental predictors of adolescent DE were explored. Associations between girls’ DE and their parents’ DE were found, and DE was also associated with difficulties with emotion dysregulation among adolescents as well as parents. Furthermore, associations were found among adolescent and parental emotion dysregulation, although the specifics of this relationship showed gender differences such as the parents’ emotion dysregulatory strategies were associated with impulse control and emotional goals for girls but not for boys. Regarding the boys, the parents’ scores on the emotional clarity subscale were associated with emotional clarity, strategies and impulse control. These associations were not found among girls. Parents’ non-acceptance scores were also positively associated with the non-acceptance scores of the boys’, but not the girls’. Only the shared family dinner meal was associated with less problems with DE and emotion dysregulation among adolescents, and finally, only parental eating disorder enhanced the probability of adolescent DE.

One boy and 22 girls reported 2 or more “yes” answers on the SCOFF, which is indicative of an eating disorder. However, only one answer on SCOFF might, in some cases, be indicative of DE [16] and about 13.1% of the boys and 35.2% of the girls reported 1 or more “yes” answers on SCOFF. These numbers are roughly comparable to studies where the prevalence of DE varied from 15 to 17% among boys and to 33 to 52% among girls in German and Finnish adolescent samples [17, 18].

Adolescents were more likely to experience DE than were their parents, which is in line with research saying that more than 50% of teenage girls and 33% of teenage boys are using restrictive measures to lose weight at any given time [33] while “only” 13 and 11% of women and men, respectively, scored in the DE range [6]. A possible explanation as to why DE is more common among younger people is that emotion regulation skills develop well into adulthood, and therefore might not be as advanced in adolescence as in later life [38]. Thus, if emotion dysregulation is indeed associated with DE, greater emotion regulation skills might be one contributor to healthier eating behaviors as a person gets older.

Girls’ DE was associated with their parents DE, which is in line with earlier research [47], and on a speculative note, considering the majority of the parents were mothers, a gender link between daughters’ and mothers’ DE could be possible, as well as a parallel relationship between boys’ and fathers’ DE. Furthermore, the girls showed greater problems with DE than did their parents, but boys did not. The number of boys who reported DE was small, and it is possible that the SCOFF is interpreted in different ways by girls and boys [16].

Girls in the study showed higher emotion dysregulation than boys, and adolescents in general had higher emotion dysregulation scores than did their parents. As mentioned earlier, the difference between adolescents and parents could be explained by prior findings saying that emotion regulation develops well into adulthood [38] but this does not explain why girls in this study experience more emotion dysregulation than boys. On the contrary, girls, in general, tend to mature earlier than boys do [27], which ought to give them an advantage in terms of emotion regulation skills. On the other hand, adolescent girls, compared to adolescent boys, are more likely to suffer from low self-esteem and depression [39], which are correlated with DE [10, 24]; these might in some way inhibit the development of emotion regulation skills among girls.

Both girls’ and boys’ emotion regulation skills were related to their parents’, which is in line with the modeling hypothesis proposed earlier [2]. According to this hypothesis, children imitate their parents’ manner of regulating their emotions implicitly. Although such modeling may explain the relations of overall DE and emotion dysregulation among adolescents and parents, it might not explain why the specific parental emotion dysregulation *strategies* were associated with adolescents’ emotion dysregulation. More specifically, this association suggests that parents have an explicit *strategy* to teach the child his or her emotion regulation skills [32].

In line with previous studies [9, 23, 30], an association between DE and emotion dysregulation was found in adolescents as well as their parents, however, parental emotion dysregulation was not associated with their children’s DE. Furthermore, there was no association between frequency of shared family meals and DE among adolescents [26]. We did observe, however, that frequency of shared family *dinners* was negatively associated with adolescent DE and emotion dysregulation, although this association was very weak. This could be because dinners are more relevant than are other meals, and might be considered more of a “family affair” in Sweden, where lunch is provided at school (and, incidentally, skipped by about 50% of adolescents) [20] and breakfast, if not completely disregarded [20], often is a stressful affair for most families with children in school and two parents working full time, although frequency of breakfast meals was earlier found to be associated with DE [1]. Perhaps “family” time is more important for reducing DE as opposed to mere “food intake,” which occurs during breakfast and lunch as well, and this might also be true for the development of emotion regulation. Furthermore, it should be noted that we only measured adolescents’ reports of how many times per week the family came together for different meals. It is possible that they have different views on what should constitute a “shared family meal” than their parents would have. It is also important to notice that not all shared meals would be considered joyful occasions, and we did not consider the wider family constellation, including siblings. A somewhat surprising result was that parental eating disorder was the only variable that raised the odds of adolescent DE. However, this result might further strengthen the importance of modeling among adolescents and their parents [2].

### Strengths and limitations

Although parents’ response rate was poor and there was a possible volunteer bias, the knowledge of which parent (mother or father) answered the questions and our matching of the parent to his or her child can be considered strengths of this study. The data were collected in common public schools in a municipality in southern Sweden and the students came from both urban and rural settings, making our sample similar to the composition in other Swedish municipalities. The final sample comprised 235 adolescent-parent pairs, which is a rather large sample overall. All data were obtained via self-report and no diagnostic interviews were used to confirm answers to the questionnaires which can be considered a limitation of the study. On the other hand, it has been argued that the anonymous self-report format might yield more valid data than interviews because they are less personally intrusive [12]. Another limitation is that, although the reliability of the DERS scale seemed appropriate for adolescents (based on the Cronbach’s alpha), it was much lower for parents. If the “awareness” subscale was removed, the Cronbach’s alpha for parents would become equivalent to that of adolescents which should be considered in further studies. The initial psychometric testing of the DERS scale, which yielded high internal consistency, was made on undergraduate students [15], and when using adult samples, the awareness subscale has been found to fail to correlate with the four of the five other subscales, while the remaining five all correlate significantly with one another [44, 45]. The fact that the DERS did not work sufficiently for the adults was a limitation, but there was an obvious advantage of using the exact same scale for the adolescents and parents. As a final limitation, we did not measure adolescents’ possible eating disorder and our question on shared meals did not contain a “never” answer.

## Conclusions and further directions

Although evidence for parental influence on DE and emotion dysregulation is inconsistent [22, 47], likely due to methodological differences [34], the present study contributes to the research by showing significant associations between parents and their adolescent children with regard to DE and emotion dysregulation as well as shared meals. Future studies should specifically ask for answers from both parents separately to further clarify the specific influences mothers and fathers have on their children. Studies should also continually include adolescents of both genders, as already suggested by Berge et al. [5]. On this note, it would also be of interest to focus on boys with DE to further explore how boys’ DE relates to their parents’ DE and emotion dysregulation. Another avenue to explore is fathers’ possible influence on their sons’ and daughters’ eating, which would offer a contrast to studies focusing solely on mothers and their responsibility for DE [11, 13], which seems to be quite outdated in contemporary society.

## Abbreviation

DE: Disordered eating
DERS: Difficulties in emotion regulation scale
